# Full-Genome Sequence of Thalassotalea euphylliae H1, Isolated from a Montipora capitata Coral Located in Hawai’i

**DOI:** 10.1128/MRA.01244-18

**Published:** 2018-11-21

**Authors:** Stephen Summers, Marnie L. Freckelton, Brian T. Nedved, Scott A. Rice, Michael G. Hadfield

**Affiliations:** a Singapore Centre for Environmental Life Sciences Engineering, Nanyang Technological University, Singapore, Singapore; b University of Hawai’i, Kewalo Marine Laboratory, Honolulu, Hawai’i, USA; c School of Biological Sciences, Nanyang Technological University, Singapore, Singapore; University of Maryland School of Medicine

## Abstract

The isolate of Thalassotalea euphylliae H1 was collected from the surface of a Montipora capitata coral. The genome was assembled using long reads from a Nanopore MinION sequencer for scaffolding and complemented with short-read MiSeq sequences. The genome was approximately 4.77 Mb long with 4,020 protein-coding genes, 92 tRNAs, and 22 rRNAs.

## Abstract (precis)

The isolate of Thalassotalea euphylliae H1 was collected from the surface of a Montipora capitata coral. The genome was assembled using long reads from a Nanopore MinION sequencer for scaffolding and complemented with short-read MiSeq sequences.

## ANNOUNCEMENT

Thalassotalea euphylliae is a Gram-negative rod-shaped bacterium originally isolated from the coral Euphylliae glabrescens (1). This bacterium is motile, having a single polar flagellum, and forms a pale-yellow colony. Members of the genus *Thalassotalea* have been isolated from the coral E. glabrescens (2), as well as from oyster tissues (3), tidal flats (4), marine sediments (5), and seawater (6).

Here, we present the genome of Thalassotalea euphyllie H1, isolated from the tissues of a coral, Montipora capitata. The original coral was collected from the reef adjacent to the Hawai’i Institute of Marine Biology (University of Hawai’i) in Kaneohe Bay, O’ahu, Hawai’i, in 2010 (7). A branch of the coral was placed in a microcentrifuge tube with 1 ml of sterile seawater and vortexed to dislodge surface bacteria, which were isolated on seawater-tryptone agar (8). The culture has been retained at –80°C at the Kewalo Marine Laboratory (University of Hawai’i, Honolulu, HI).

For this study, the isolate was grown overnight at 28°C in a seawater-tryptone broth (8). Following incubation, cells were pelleted by centrifugation at 4,000 × *g* for 5 min. Nucleic acids were extracted using a xanthogenate-based extraction technique (9). Following extraction, long-read sequencing was performed on a Nanopore MinION genome sequencer using a rapid sequencer kit (catalog number SQK-RAD004) and an R9.4 flow cell. This resulted in 444,735 reads of up to 116,180 bp long (mean, 5,554 bp), which were used as a scaffold for the genome assembly. To aid in the assembly, 300-bp paired-end sequencing was carried out on the Illumina MiSeq platform after library preparation using a TruSeq Nano DNA kit, which yielded 874,001 reads.

The long reads were base called using Albacore v2.3.1 (default settings), as supplied by Nanopore, and adapters were removed using Porechop v0.2.3 (verbosity level 3). The *de novo* assembly was conducted using Unicycler v0.4.4 (10); MiSeq sequences were used for short-read input (forward and reverse), and MinION reads were used for long-read input, with polishing and rotation conducted by Racon v1.3.1 (11) using default settings.

The final sequence was 4,773,423 bp long (144-fold coverage, 44.3% GC content) and formed a contiguous, circular chromosome. Species identification was achieved by BLASTn comparison of the NCBI 16S rRNA gene database and showed a 96% match to *T. euphylliae* strain Eup-16 (GenBank accession number NR_153727).

Annotation with the NCBI Prokaryotic Genome Annotation Pipeline (PGAP) v4.2 (12) identified 4,138 genes consisting of 4,020 protein-coding genes and 118 RNAs (92 tRNAs, 4 noncoding RNAs, 8 5S rRNA subunits, and 7 copies each of the 16S and 23S rRNAs). Using annotation services provided by the Rapid Annotations using Subsystems Technology (RAST [13]) servers, we identified 4,347 DNA coding sequences within 323 subsystems. In addition, 3 CRISPR arrays (2,359, 2,467, and 1,637 bp), 104 CRISPR repeats, and 102 CRISPR spaces were identified.

### Data availability.

The full-genome sequence of *T. euphylliae* H1 has been deposited in GenBank (accession number QUOU00000000). Raw sequencing reads are available in the NCBI Sequence Read Archive (accession numbers SRR8069227 and SRR8069226).
